# Correlations between Low Doses of Zearalenone, Its Carryover Factor and Estrogen Receptor Expression in Different Segments of the Intestines in Pre-Pubertal Gilts—A Study Protocol

**DOI:** 10.3390/toxins13060379

**Published:** 2021-05-26

**Authors:** Magdalena Gajęcka, Magdalena Mróz, Paweł Brzuzan, Ewa Onyszek, Łukasz Zielonka, Karolina Lipczyńska-Ilczuk, Katarzyna E. Przybyłowicz, Andrzej Babuchowski, Maciej T. Gajęcki

**Affiliations:** 1 Department of Veterinary Prevention and Feed Hygiene, Faculty of Veterinary Medicine, University of Warmia and Mazury in Olsztyn, Oczapowskiego 13, 10-718 Olsztyn, Poland; magdzia.mroz@gmail.com (M.M.); lukaszz@uwm.edu.pl (Ł.Z.); gajecki@uwm.edu.pl (M.T.G.); 2 Department of Environmental Biotechnology, Faculty of Environmental Sciences and Fisheries, University of Warmia and Mazury in Olsztyn, Słoneczna 45G, 10-719 Olsztyn, Poland; brzuzan@uwm.edu.pl; 3 Dairy Industry Innovation Institute Ltd., Kormoranów 1, 11-700 Mrągowo, Poland; ewa.onyszek@iipm.pl (E.O.); andrzej.babuchowski@iipm.pl (A.B.); 4 Department of Epizootiology, Faculty of Veterinary Medicine, University of Warmia and Mazury in Olsztyn, Oczapowskiego 13/01, 10-718 Olsztyn, Poland; karolina.lipczynska@uwm.edu.pl; 5 Department of Human Nutrition, Faculty of Food Sciences, University of Warmia and Mazury in Olsztyn, Słoneczna 45F, 10-719 Olsztyn, Poland; katarzyna.przybylowicz@uwm.edu.pl

**Keywords:** zearalenone, digestive tract, carryover factor, ERs *m*RNA, CYP1A1 *m*RNA, GSTP1 *m*RNA, pre-pubertal gilts

## Abstract

Plant materials can be contaminated with *Fusarium* mycotoxins and their derivatives, whose toxic effects on humans and animals may remain subclinical. Zearalenone (ZEN), a low-molecular-weight compound, is produced by molds in crop plants as a secondary metabolite. The objective of this study will be to analyze the in vivo correlations between very low monotonic doses of ZEN (5, 10, and 15 μg ZEN/kg body weight—BW for 42 days) and the carryover of this mycotoxin and its selected metabolites from the intestinal contents to the intestinal walls, the *m*RNA expression of estrogen receptor alfa (ER*α*) and estrogen receptor beta (ER*β*) genes, and the *m*RNA expression of genes modulating selected colon enzymes (CYP1A1 and GSTP1) in the intestinal mucosa of pre-pubertal gilts. An in vivo experiment will be performed on 60 clinically healthy animals with initial BW of 14.5 ± 2 kg. The gilts will be randomly divided into a control group (group C, *n* = 15) and three experimental groups (group ZEN5, group ZEN10, and group ZEN15; *n* = 15). Group ZEN5 will be administered *per os* 5 μg ZEN/kg BW (MABEL), group ZEN10—10 μg ZEN/kg BW (NOAEL), and group ZEN15—15 µg ZEN/kg BW (low LOAEL). In each group, five animals will be euthanized on analytical dates 1 (exposure day 7), 2 (exposure day 21), and 3 (exposure day 42). Samples for in vitro analyses will be collected from an intestinal segment resected from the following regions: the third (horizontal) part of the duodenum, jejunum, ileum, cecum, ascending colon, transverse colon, and descending colon. The experimental material will be collected under special conditions, and it will be transported to specialist laboratories where samples will be obtained for further analyses.

## 1. Introduction

A high percentage of plant-based raw materials used in feed production [1] may be contaminated with mycotoxins (undesirable substances), thus increasing the risk of poisoning in humans [2] and farm animals, particularly pigs [3]. The toxicological effects, health risks, and symptoms associated with exposure to high levels of mycotoxins, including zearalenone (ZEN), have been well documented [4,5,6,7]. According to the hormesis paradigm [8,9], health implications of exposure to low (measurable) doses of mycotoxins that are frequently encountered in feedstuffs are becoming increasingly important and need to be investigated. The dysfunctions caused by exposure to pure parent compounds [10,11,12] without metabolites or modified mycotoxins [9] constitute an interesting object of study in mammals.

Fusarium mycotoxins are absorbed primarily in the proximal part of the small intestine [3,13] due to considerable physiological differences between intestinal segments. Mycotoxins are transported to the blood, and blood analyses support noninvasive diagnoses of animal health [14] and the identification of new biomarkers of disease [15]. For instance, ZEN could be regarded as a pharmacodynamic biomarker [16] for determining, at least partially, the interactions between the mycotoxin and its target [17]. Zearalenone and its metabolites usually target cells [18], where they induce specific changes by regulating enzyme metabolism [9,19], gene expression [12], and by acting as ligands that bind to specific receptors in cell membranes and cell nuclei [20]. These mechanisms enable mycotoxins to participate in signal transduction, including in gastrointestinal tract tissues. 

The existing body of knowledge [21] suggests that the side effects of exposure to low ZEN doses may be difficult to predict. This uncertainty results not only from the ingested dose but also from the time of exposure [15]. Low levels of mycotoxins can induce unexpected responses. For example, the presence of those undesirable substances may be “ignored” by the body or remain [22], in accordance with the theory of regulatory T-cells (T-regs) that states that they do not respond to a low number of infectious factors in the organism [23]. During prolonged exposure to ZEN administered *per os*, its absorption increases in the host organism [24], which is accompanied by a compensatory effect [25], where the activity of the analyzed parameters is initially suppressed and then returns to baseline values [26] despite ongoing exposure [10]. The above factors, the multidirectional effects of low doses of ZEN and its metabolites [21] in the porcine diet, as well as their ability to elicit specific responses in gilts require further scientific inquiry.

In our previous research [10,11,12], a low dose was defined [16] based on the presence or absence of clinical symptoms of ZEN mycotoxicosis. Three variants of mycotoxin doses were proposed based on our results and the findings of other authors: the lowest observed adverse effect level (LOAEL) dose (>10 μg ZEN/kg BW) [21,27,28] that induces clinical symptoms [3], the no observed adverse effects levels (NOAEL) dose (10 μg ZEN/kg BW), i.e., the highest dose that does not produce clinical symptoms (subclinical states) [29], and the minimal anticipated biological effect level (MABEL) dose (<10 μg ZEN/kg BW) [16], which is the smallest measurable dose (or the effective dose in vivo) that enters into positive interactions with the host in different life stages [7,10,11,12,16,30,31].

Since ZEN is a mycoestrogen, the existing dose–response paradigm can be superseded by the low-dose hypothesis [21], in particular with regard to hormonally active compounds [32]. This ambiguous dose–response relationship does not justify a simple and definitive translation of the risk associated with high doses to low doses [3,26] that are effective in vivo. The concept of a minimal dose has gained increasing popularity in biomedical sciences [14,16], and it is consistent with the main tenets of precision medicine [33]. This breakthrough concept accounts for individual variations as well as population characteristics to explain the holistic effects of a minimal dose. Such an approach expands the existing biological knowledge and examines individual variations (in livestock farming) in different areas of biological and medical sciences. Such analyses rely on specialist laboratory equipment to acquire comprehensive information about biomarkers (genes, gene expression products, and metabolites) [14,34]. However, a low dose can also be defined as a dose that delivers counterintuitive effects [8,16]. Therefore, the mechanisms of action associated with a low dose have to be understood to facilitate decision-making in quantitative and qualitative risk assessments [35,36].

Zearalenone metabolites are usually not detected during exposure to low doses of this mycotoxin. According to most studies, more α-ZEL than β-ZEL is produced during ZEN biotransformation in pigs, which are characterized by higher activity levels of 3α-HSD than that of other animal species [37]. However, this is not always the case, as demonstrated by a study conducted in our research center [38]. In pre-pubertal gilts, the peripheral blood concentrations of ZEN and its metabolites point to ongoing biotransformation processes that do not lead to hyperestrogenism, but compensate for a physiological deficiency of endogenous estrogens [39]. These observations have been confirmed by an analysis of the concentrations of the parent compound and its metabolites in gilts exposed to MABEL doses of ZEN [40]. Zearalenone metabolites were not identified in the first week of exposure, probably due to an inadequate supply of endogenous steroid hormones.

In contrast to the findings of other authors [37,40] and our previous research [21], β-ZEL was the predominant metabolite in the blood serum of gilts in successive weeks of exposure [38]. It could be speculated that the host organism tries to compensate for the physiological deficiency of endogenous estrogens (which are necessary for the essential life processes) [41], and its demand for compounds with high estrogenic activity (such as α-ZEL and ZEN) increases [40,42]. It is also possible that the seventh day of exposure to an undesirable substance such as ZEN marks the end of adaptive processes (adaptive immunity) [43]. Zearalenone could also be utilized as a substrate that regulates (in an inversely proportional manner) the expression of genes encoding HSDs—molecular switches modulating steroid hormone prereceptors [19,44,45]. Undesirable substances could also undergo enterohepatic recirculation before they are eliminated from the body [21,46], and/or exposure to ZEN could induce a specific response from the distal gut microbiota [11].

The above hypotheses, alone or in combination, could explain the peripheral blood concentrations of ZEN and its metabolites because this mycotoxin exerts multidirectional effects [21]. Zearalenone inhibits the synthesis and secretion of the follicle-stimulating hormone (FSH) [37] via negative feedback, thus decreasing steroid production [47,48].

During prolonged exposure to low ZEN doses, similar interdependences are observed between the average concentrations of ZEN and its metabolites in peripheral blood [37]. However, two differences were observed during prolonged (longer than seven days) exposure to ZEN. First, both ZEN metabolites were detected in peripheral blood. Next, the values of all evaluated parameters increased, probably due to the accumulation of ZEN and its metabolites (resulting from the saturation of active ERs and other factors that influence the levels of steroid hormones [47,48,49]), over time. In other studies investigating the effects of ZEN biotransformation in peripheral blood, animals were exposed to much higher doses of the mycotoxin [21,50,51]. These observations could suggest that in line with the hormesis paradigm [8,21], the exposure to very low doses of ZEN affects the synthesis and secretion of sex steroid hormones [18,39]. Therefore, the simultaneous processes of the biotransformation of very low ZEN doses are not identical, and the parent compound (ZEN) and its metabolites are utilized either completely or to a much larger extent (which was also the case during exposure to the MABEL dose) by the host organism [16]. The interactions between endogenous and exogenous (environmental) steroids could also be influenced by other endogenous factors, such as the accumulation of ZEN and its metabolites in intestinal tissues in the initial stages of biotransformation, the resulting expression of ERs and selected intestinal enzymes that participate in detoxification, which is accompanied by specific (but not clearly determined) accumulation of ZEN and its metabolites in intestinal tissues.

Undesirable substances such as ZEN are metabolized inside cells by two classes of enzymes. Phase I enzymes modify undesirable substances via several processes, including hydroxylation. These enzymes are known as cytochromes (CYP), they are abundant in the body and tissue-specific. Phase II enzymes, such as glutathione S-transferase (GST), conjugate metabolites through glucuronidation [52].

The P450 cytochrome (CYP) superfamily consists of several hundred isoenzymes that catalyze the oxidation of various substrates, including exogenous (xenobiotics) and endogenous (hormones, prostaglandins, and vitamins) compounds [53]. Many CYPs are inducible, which significantly increases their catalytic activity after exposure to specific chemical substances [54]. These compounds are ligands of specific receptors, such as the aryl hydrocarbon receptor (AhR) and ERs. Activated receptors are transferred to the nucleus, they undergo dimerization with nuclear partners, bind to specific sequences in subsequent promotors, and induce the transcription of target genes [55]. The above increases *m*RNA levels and enhances the synthesis of CYP protein. This process ultimately boosts the enzymatic activity of specific CYPs [56].

In phase II, liver cells could become resistant to various substances due to the intensification of metabolic process and detoxification of undesirable compounds in feed [57]. The π isoform of glutathione S-transferase (GSTP1) is one of the molecules that elicit these types of mechanisms. In the body, GST occurs in the form of numerous isoenzymes, which have been divided into classes on the basis of their location in the cell, amino acid sequences, location of genes, and substrate specificity [58]. The role of GST is not limited to the detoxification of exogenous electrophilic toxins. The enzyme also protects the body against the harmful products of oxidative stress [59], and prevents damage to nucleic acids and lipids. Glutathione S-transferase participates in the metabolism of steroid hormones, biosynthesis of leukotriene C4 and prostaglandin E_2_, and the maintenance of glutathione homeostasis [60].

The aim of the proposed study will be to determine in vivo the correlations between very low monotonic doses of ZEN (5, 10, and 15 μg ZEN/kg BW for 42 days), the carryover of ZEN and its metabolites from the intestinal contents to the intestinal walls, the *m*RNA expression of ER*α* and ER*β* genes, and the *m*RNA expression of genes modulating selected colon enzymes (CYP1A1 and GSTP1) in the intestinal mucosa of pre-pubertal gilts. The study will expand our understanding of the mechanisms underlying the expression of ER*α* and Er*β,* which participate in both stages of mycotoxin (undesirable substance) biotransformation.

## 2. Materials and Methods

In our opinion, the research hypotheses could be effectively validated with the use of the methods deployed in precision medicine [14,33]. Precision medicine involves assessments of individual animals as well as entire populations, and the results of these observations play a very important role in clinical veterinary practice. Holistic methods have to be applied to deepen our understanding of pathological processes. These methods include technologies based on mass spectrometry or high-throughput molecular biology techniques.

### 2.1. Experimental Procedures

All procedures were carried out in compliance with Polish legal regulations for the determination of the terms and methods for performing experiments on animals and with the European Community Directive for the ethical use of experimental animals. The protocol was approved by the Local Ethics Committee for Animal Experimentation at the University of Warmia and Mazury in Olsztyn, Poland (opinion No. 42/2019 of 28 May 2019).

### 2.2. Experimental Animals and Feeding

An in vivo experiment will be performed at the Department of Veterinary Prevention and Feed Hygiene of the Faculty of Veterinary Medicine at the University of Warmia and Mazury in Olsztyn on 60 clinically healthy pre-pubertal gilts with initial BW of 14.5 ± 2 kg [10]. During the experiment, the animals will be housed in pens, they will be fed identical diets, and water will be available ad libitum. The gilts will be randomly divided into a control group (group C; *n* = 15) and three experimental groups (ZEN5, ZEN10, and ZEN15; *n* = 15 each) [61,62]. Groups ZEN5, ZEN10, and ZEN15 will be administered ZEN (Sigma-Aldrich Z2125-26MG, St. Louis, MO, USA) *per os* at 5 μg/kg BW, 10 μg/kg BW, and 15 μg/kg BW, respectively. Each experimental group will stand in a separate pen and in the same building. The pens have an area of 25 m^2^, which complies with the applicable cross compliance regulations (Regulation (EU) No 1306/2013 of the European Parliament and of the Council of 17 December 2013).

Analytical samples of ZEN will be dissolved in 96 μL of 96% ethanol (SWW 2442-90, Polskie Odczynniki SA, Poland) in weight-appropriate doses. Feed containing different amounts of ZEN in an alcohol solution will be placed in gel capsules. Before administration, the capsules will be stored at room temperature until the alcohol evaporates. Experimental group pigs will receive ZEN in gel capsules every day before morning feeding. The animals will be weighed at weekly intervals in order to adjust individual mycotoxin doses. Feed will be the carrier, and group C gilts will receive identical gel capsules without ZEN [10,11]. The feed will be supplied by the same producer. During the experiment, the gilts will be offered feed in friable form ad libitum twice daily (at 8:00 a.m. and 5:00 p.m.). The ingredient composition of complete diets will be specified by the manufacturer, as presented in Table 1.

The proximate chemical composition of the diets fed to gilts in groups C, ZEN5, ZEN10, and ZEN15 will be analyzed with the NIRS-DS2500 F monochromator-based NIR reflectance and transflectance analyzer with a scanning range of 850–2500 nm (FOSS, Hillerød, Denmark).

### 2.3. Toxicological Analyses

#### 2.3.1. Determination of Mycotoxins in Feed

Feed will be analyzed for the presence of ZEN and DON (deoxynivalenol), and their concentrations will be determined by separation in immunoaffinity columns (Zearala-TestTM Zearalenone Testing System, G1012, VICAM, Watertown, MA, USA; DON-TestTM DON Testing System, VICAM, Watertown, MA, USA) and high-performance liquid chromatography (HPLC system, Agilent 1260)–mass spectrometry (MS, Agilent 6470) and chromatography columns (Atlantis T3 3 μm 3.0 150 mm Column No. 186003723, Waters, AN Etten-Leur, Ireland). Mycotoxins will be separated using a mobile phase of acetonitrile:water:methanol (46:46:8, *v*/*v*/*v*). The flow rate will be 0.4 mL/min. The limit of quantitation (LoQ) for ZEN will be 2 ng/g and 5 ng/g for DON. Zearalenone and its metabolites will be quantified at the Department of Veterinary Prevention and Feed Hygiene [63].

#### 2.3.2. Biotransformation of ZEN

##### Tissue Samples

In each group, 5 animals will be euthanized on analytical dates 1 (D1—exposure day 7), 2 (D2—exposure day 21), and 3 (D3—exposure day 42) by the intravenous administration of pentobarbital sodium (Fatro, Ozzano Emilia BO, Italy) and bleeding. Immediately after cardiac arrest, tissue samples (approximately 1 × 1.5 cm) will be collected from entire intestinal cross-sections, from the following segments of the gastrointestinal tract: the duodenum—the first part (duodenal cap) and the horizontal or third part; the jejunum and ileum—middle parts; colon—middle parts of the ascending colon, transverse colon, and descending colon; the cecum—1 cm from the ileocecal valve. The samples will be rinsed with phosphate buffer and prepared for analyses [5,6].

##### Extraction and Purification

Zearalenone, α-ZEL, and β-ZEL will be extracted from tissues with the use of immunoaffinity columns (Zearala-TestTM Zearalenone Testing System, G1012, VICAM, Watertown, MA, USA) according to the manufacturer’s recommendations. The obtained eluents will be placed in a water bath at 50 °C, and will be evaporated in a stream of nitrogen. The dry residue will be stored at –20 °C until chromatographic analysis. The procedure will be monitored with the use of internal standards, and the results will be validated by mass spectrometry.

##### Chromatographic Determination of the Concentrations of Zen and its Metabolites

The tissue concentrations of ZEN, α-ZEL, and β-ZEL will be determined with the Agilent 1260 liquid chromatograph (LC) and the Agilent 6470 mass spectrometer (MS). The prepared samples will be analyzed with the use of the Zorbax rapid resolution chromatographic column (2.1 × 50 mm; 1.8 micron Agilent Eclipse Plus C18) in gradient mode. The mobile phase will contain 0.1% (*v*/*v*) formic acid in water (solvent A) and 0.1% (*v*/*v*) formic acid in acetonitrile (solvent B). Gradient conditions will be as follows: initially, 20% B that increases to 100% B in 4.0 min and back to 20% B in 0.1 min.

Mycotoxin concentrations will be determined according to an external standard and will be expressed in ppb (ng/mL). The quantification process will involve matrix-matched calibration standards to eliminate matrix effects that can decrease sensitivity. Calibration standards will be dissolved in matrix samples based on the procedure described for the remaining samples. The material for preparing calibration standards will be free of mycotoxins. A signal-to-noise ratio of 3:1 will be used to estimate the limits of detection (LOD) for ZEN, α-ZEL, and β-ZEL. The LOQ will be estimated as the triple LOD value.

The specificity of the method will be determined by comparing the chromatograms of a blank sample with those corresponding to a spiked tissue sample.

##### Mass Spectrometric Conditions

The mass spectrometer was operate with ESI in the negative ion mode. The MS/MS parameters were opimized for each compoud. The linearity was tested by a calibration curve including six levels. Table 2 shows the optimized analysis conditions for the mycotoxins tested.

##### Statistical Analysis

The results of the study will be processed at the Department of Discrete Mathematics and Theoretical Computer Science at the Faculty of Mathematics and Computer Science of the University of Warmia and Mazury in Olsztyn. The bioavailability of ZEN and its metabolites in the intestinal tissues of pre-pubertal gilts will be analyzed in three experimental groups and the control group, on different sampling dates. The results will be expressed as mean values ($\bar{x}$) and standard deviation (SD). The following tests will be carried out: (i) analyses of differences between the mean values in three experimental groups (receiving different doses of ZEN) and the control group on three analytical dates; (ii) analyses of differences between the mean values within groups (receiving the same ZEN dose) on each analytical date. In both tests, differences between mean values will be determined by one-way ANOVA. If the differences between groups are statistically significant, differences between pairs of means will be estimated by Tukey’s multiple comparison test. If all values are below LOD (mean and variance are equal to zero) in any group, the values in the remaining groups will be processed by one-way ANOVA, and the differences between means in these groups will be compared with the population mean difference of zero in Student’s t-test. Differences between groups will be estimated by Student’s t-test. The results of each analysis will be considered to be highly significant at *p* < 0.01 (**) and significant at 0.01 < *p* < 0.05 (*). Data will be analyzed in the Statistica v.13 program (TIBCO Software Inc., Silicon Valley, CA, USA, 2017).

### 2.4. Expression of ERα, ERβ, CYP1A1, and GSTP1 Genes

#### 2.4.1. Collection and storage of samples for RNA Extraction

Immediately after cardiac arrest, tissue samples will be collected from the duodenum—the first part (duodenal cap) and the horizontal or third part; the jejunum and ileum—middle parts, and the colon—middle parts of the ascending colon, transverse colon, and descending colon. The samples will be stored in RNA*later* solution (Sigma-Aldrich; Germany), in accordance with the manufacturer’s instructions. Tissue samples will be collected on the same dates.

#### 2.4.2. Total RNA Extraction and CDNA Synthesis

Total RNA will be extracted from the tissues preserved in RNA*later* (approximately 20 mg per sample; *n* ¼ 3 in each experimental group) using the Total RNA Mini isolation kit (A&A Biotechnology; Poland) according to the manufacturer’s protocol. RNA samples will be incubated with RNase-free DNase I (Roche Diagnostics; Germany) to prevent contamination of genomic DNA. Total RNA quality and the purity of all samples will be estimated with the BioPhotometer (Eppendorf; Germany), and the results will be used to synthesize cDNA with the RevertAid™ First Strand cDNA Synthesis Kit (Fermentas; Canada). The cDNA synthesis reaction mixture for each sample will contain 1 μg of total RNA and 0.5 μg of oligo (dT)18 primers, and the reaction will be performed according to the manufacturer’s protocol. The first synthesized cDNA strand will be stored at −20 °C for further analysis.

#### 2.4.3. qPCR

Real-time PCR primers for ER*α* and ER*β* mRNAs, and CYP1A1 and GSTP1 mRNAs will be designed using the Primer-BLAST tool based on the reference species (Table 3). *β*-actin will be used as the endogenous reference gene. The real-time PCR assay will be performed in the ABI 7500 real-time PCR system thermocycler (Applied Biosystems, Foster City, CA, USA) in singleplex mode. Further treatments will be applied as recommended by the manufacturer.

Quantitative cycle (Cq) values from qPCR will be converted to copy numbers using a standard curve plot (Cq versus log copy number) according to the methodology.

The rationale for using the standard curve is based on the assumption that unknown samples have equal amplification efficiency (usually above 90%), which is checked before unknown standards are extrapolated to the standard curve. To generate the standard curves, purified PCR products of each mRNA will be used to prepare a series of six 10-fold dilutions with known amounts of copy numbers, which will be used as templates in real-time PCR. The Cq values obtained for each dilution series will be plotted against the log copy number, and will be used to extrapolate unknown samples to copy numbers. mRNA copy numbers of the samples collected from all experimental groups in each exposure period will be divided by the averaged numbers from the control group, determined at the beginning of the experiment (control 0d), to obtain relative expression values, which will be presented as the expression ratio (R).

#### 2.4.4. Statistical Analysis

The expression of ERα and ERβ in the digestive tract of gilts, and the expression of *CYP1A1* and *GSTP1* genes in the ascending colon and the descending colon will be presented as mean values (±) SD for each sample. The results will be analyzed using Statistica software (StatSoft Inc., USA). The mean values in the control and experimental groups will be compared by repeated-measures one-way ANOVA based on the ZEN dose administered to pre-pubertal gilts. If differences between groups are found, Tukey’s post hoc test will be performed to determine which pairs of group means are significantly different. In ANOVA, group samples will be drawn from normally distributed populations characterized by the same variance. If the above assumptions are not met in all cases, the equality of group means will be tested using the Kruskal–Wallis test of ranks and the multiple comparisons test in ANOVA. Different group pairs will be identified by post hoc multiple comparisons of mean ranks for all groups.

## 3. Discussion

Mycotoxins have always been and will always be present in foodstuffs and feedstuffs—this is a banal truth. Higher mycotoxin doses can produce symptoms of mycotoxicosis (poisoning) in macroorganisms. However, little is known about the fate of mycotoxins and the responses of the host organism during exposure to low doses of mycotoxins in the range of NOAEL and MABEL doses [16]. Macroorganisms have developed various coping strategies that enable them to maintain homeostasis. These coping mechanisms can involve tolerance to very low mycotoxin doses [16]. Alternatively, mycotoxins can participate in vital life processes, as briefly noted in the Introduction. Mycotoxins are accumulated and absorbed not only in target tissues [66,67], which suggests that the intestinal mucosa containing ERs is the most exposed tissue and the first line of defense against undesirable substances [68]. Therefore, the relevant diagnostic tests and laboratory analyses will be performed in the proposed study.

Due to the general scarcity of published research into low-dose mycotoxicosis, additional in vivo data are needed to increase the safety of foodstuffs and feedstuffs, and minimize the risk of toxicity in the decision-making process [69]. The proposed study will attempt to determine whether low doses of ZEN can affect (i) the degree and site of β-ZEL accumulation in the porcine gastrointestinal tract at different exposure times; (ii) the expression of ERs in the porcine gastrointestinal tract, analyzed in vivo, in particular, the expression of Er*α,* which regulates intestinal function in the proximal segments of the gastrointestinal tract; (iii) the involvement of intestinal enzymes (expression effect) in the distal segment of the intestines in ZEN detoxification processes.

The results of the study will support the development of biomarkers of prolonged low-dose ZEN mycotoxicosis in pre-pubertal gilts within the framework of veterinary precision medicine.

## Figures and Tables

**Figure 1:**
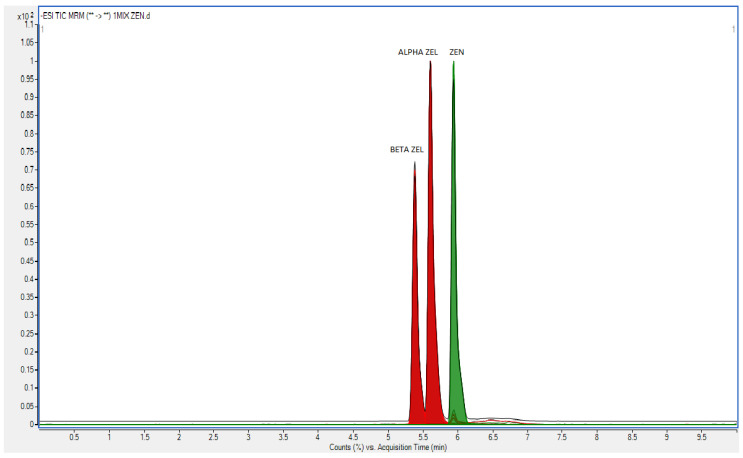
The chromatogram of standard solution.

**Table 1:** Declared composition of the complete diet.

Parameters	Composition Declared by the Manufacturer (%)
Soybean meal	16
Wheat	55
Barley	22
Wheat bran	4.0
Chalk	0.3
Zitrosan	0.2
Vitamin–mineral premix ^1^	2.5

^1^ Composition of the vitamin–mineral premix per kg: vitamin A—500.000 IU; iron—5000 mg; vitamin D3—100.000 IU; zinc—5000 mg; vitamin E (alpha-tocopherol)—2000 mg; manganese—3000 mg; vitamin K—150 mg; copper (CuSO_4_·5H_2_O)—500 mg; vitamin B1—100 mg; cobalt—20 mg; vitamin B2—300 mg; iodine—40 mg; vitamin B6—150 mg; selenium—15 mg; vitamin B12—1500 μg; L-lysine—9.4 g; niacin—1200 mg; DL—methionine + cystine—3.7 g; pantothenic acid—600 mg; L-threonine—2.3 g; folic acid—50 mg; tryptophan—1.1 g; biotin—7500 μg; phytase + choline—10 g; ToyoCerin probiotic + calcium—250 g; antioxidant + mineral phosphorus and released phosphorus—60 g; magnesium—5 g; sodium and calcium—51 g.

**Table 2:** Optimaized conditions for mycotoxins tested.

Analyte	Precursor (*m*/*z*)	Production (*m*/*z*)	FragmentorVoltage (V)	Collision Energy (eV)	LOD (ng mL^−1^)	LOQ (ng mL^−1^)	Linearity (%R^2^)
ZEN	317.1	273.3 187.1	160	25 33	0.03	0.1	0.999
*α*-ZEL	319.2	275.2 160.1	144	21 33	0.3	0.9	0.997
*β*-ZEL	319.2	275.2 160.1	144	21 33	0.3	1	0.993

A chromatogram of standard mixtures of all analytes is presented in Figure 1.

**Table 3:** Real-time PCR primers for the proposed study.

Primer	Sequence (5′→3′)		Amplicon Length (bp)	References
ERALFA	Forward	Agggaagctcctattgctcc	234	[64]
	Reverse	cggtggatgtggtccttctct		
ERBETA	Forward	Gcttcgtggagctcagcctg	262	[64]
	Reverse	aggatcatggccttgacacaga		
CYP1A1	Forward	cagagccgcagcagccaccttg	226	[48]
	Reverse	ggctcttgcccaaggtcagcac		
GSTP1	Forward	acctgcttcggattcaccag	178	[48]
	Reverse	ctccagccacaaagccctta		
β-Actin	Forward	catcaccatcggcaaaga	237	[65]
	Reverse	gcgtagaggtccttcctgatgt		

## Data Availability

Not applicable.
